# Child Sexual Abuse among School Children of a Municipality: A Descriptive Cross-sectional Study

**DOI:** 10.31729/jnma.6865

**Published:** 2021-07-31

**Authors:** Sushma Shrestha, Swechhya Baskota, Urusha Karki, Lisasha Poudel, Niroj Bhandari, Minani Gurung, Bibek Rajbhandari, Pramita Shrestha

**Affiliations:** 1 Department of Public Health, Om Health Campus, Kathmandu, Nepal; 2 Central Department Tribhuwan University, Kathmandu, Nepal; 3 Dhulikhel Hospital, Kathmandu University School of Medical Sciences, Kavre, Nepal; 4 Kathmandu University School of Medical Sciences, Kavre, Nepal; 5 One Health Research and Training Center, Kathmandu, Nepal; 6 Nepal Police Hospital, Kathmandu, Nepal

**Keywords:** *child sexual abuse*, *Nepal*, *prevalence*

## Abstract

**Introduction:**

Sexual abuse remains a hidden and underreported form of violence against children and a serious public health concern. Although it results in severe mental, physical, and psychological risks with consequences such as depression, fear, and low self-esteem, it is still an unexplored and less prioritized area in Nepal. The objective of this study was to determine the prevalence of Child Sexual Abuse among school children of a municipality.

**Methods:**

This was a descriptive cross-sectional study, conducted among 405 students, aged below 18 of Mandan-Deupur Municipality, Kavrepalanchowk from April to May 2018. Ethical clearance was taken from Nepal Health Research Council (Reference no 2506). Simple random sampling technique was used to select the schools. Collected data were then entered and analyzed using Statistical Package for Social Sciences version 16. Point estimate at 95% Confidence Interval was calculated along with frequency and percentage.

**Results:**

In overall, 64 (15.8%) (12.4-19.7 at 95% Confidence Interval) were found to be sexually abused, where a high prevalence of Child Sexual Abuse was reported for boys 46 (73.43%).

**Conclusions:**

According to the findings of this study, child sexual abuse is seen as a significant issue in the place studied. Awareness programs on child sexual abuse should be organized targeting children along with parents and community people.

## INTRODUCTION

A child under the age of 1 8, if gets involved in any sexual activity that he or she does not fully comprehend is defined as Child Sexual Abuse (CSA). Such activity is evident between a child and an adult or another child who by age or development is in a relationship of responsibility, trust or power, for gratifying the needs of the other person.^1^ CSA results in severe mental, physical, and psychological hazards such as depression, fear, and low self-esteem.^2,3^ WHO reports that a child (aged 0-17 years), one in five women and one in 13 men have been sexually harassed.^4^

In Nepal, child sexual abuse is prevalent in families, neighborhoods, schools, streets, workplaces, social media and so on.^5-8^ CSA remains underexplored and less prioritized area of study.^9^

The objective of this study was to find out the prevalence of CSA among school children of Mandan-Deupur Municipality of Kavre district, Nepal.

## METHODS

We conducted a descriptive cross-sectional study among the high school students of Mandan-Deupur Municipality, Kavrepalanchowk, Nepal. This was conducted between April to May, 2018. We obtained ethical clearance approval from the Nepal Health Research Council (NHRC approval no. 2506) and also got official permission from the school administration. We acquired written accent and consent from the parents and students respectively.

We included students below 18 years studying in grade 8, 9 and 10 of the four higher secondary schools in the municipality. We excluded those who did not gave their consent and were absent during the survey day.

Prevalence i.e. 41% was taken from a study conducted in Nepal.^10^ We determined sample size using the formula:

n = Z^2^ × p × (1 - p) / e^2^

  = (1.96)^2^ × (0.41) × (1-0.41) / (0.05)^2^

  = 372

Where,

n = required sample size
Z = 1.96 at 95% level of Confidence Interval
e = margin of error, 5%
q = 1-p
p = prevalence from the previous study, 41%^2^ Including 9% non-response rate= 9% of 372 = 33.48 ~ 33

Hence, the sample size was 405. We obtained the list of secondary schools along with student's number from Mandan-Deupur Municipality. Simple random Sampling method was use. We selected 8, 9, and 10 grade students they are in such a transition age to get victim of CSA and also a suitable age to get the selfadministered questions filled. We included 405 school student of grade 8, 9, and 10 from the selected schools.

Firstly, we contacted the teachers to orient them the study questionnaire, purpose of the study, privacy and confidentiality that will be maintained throughout the study. After the verbal permission, the students were distributed the accent and the consent sheet to get it from their parents and the students. This was followed by the distribution of the self-administered questionnaire to the participants to gather the information. In this study, we investigated socio-demographic and child sexual abuse characteristics. Questionnaires were adapted from various studies.^10-12^ We translated the questionnaires into Nepali language and experts were consulted to check the translated questionnaire. We pretested the questionnaire in 10% of calculated sample size, in a similar setting to verify the tools developed in Nepali language. We used Statistical Package for Social Sciences (SPSS) version 16 for data management and analysis. We calculated the descriptive statistics using frequencies and percentages to describe the study population and interpreted them into tables. Point estimate at 95% Confidence Interval was calculated along with frequency and percentage.

## RESULTS

**Figure 1:**
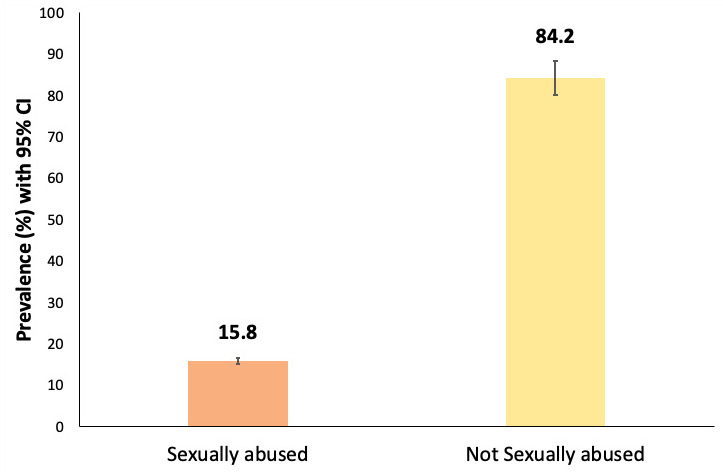
Prevalence of child abuse.

Out of 405 respondents, 64 (15.8%) (12.4-19.7 at 95% Confidence Interval) were found to be sexually abused (Figure 1), where high prevalence of CSA was among boys 47 (73.43%).

More than one third 24 (37.5%) of respondents were sexually abused when they were 12-16 years. Around 5 (7.81%) of them were sexually abused before their age was 6 years. CSA was highly prevalent among boys 47 (73.43%) rather than girls (26.56%) (Table 1). Below table reveals that among sexually abused respondents, 45 (66.2%) were forced to watch sexual activities and almost half of the victims 29 (45.31 %) were sexually abused 1 time. Most of the respondents were sexually abused at public places. All of the respondents had experienced some sort of immediate effect after being sexually abused. Out of 64 respondents who were sexually abused, only half of them disclosed the incident with their parents 2 (5.7%) and friends 33 (94.3%). In spite of disclosure, 13 (38.2%) of victims were ignored and 12 (32.4%) of them were not believed by anyone. The main reason for not disclosing about the event was because of shame 24 (44.4%) (Table 1).

**Table 1:** Prevalence of Child Sexual Abuse according to gender and age and characteristics of CSA (n = 64).

Characteristics	n (%)
**Gender**	
Female	17 (26.6)
Male	47 (73.4)
**Age of respondent (years)**	
Less than 6	5 (7.8)
7-12	19 (29.7)
12-16	24 (37.5)
**Characteristics of CSA**	
**Characteristics**	**n (%)**
**Forms of CSA** ^*^	
Forced to watch or show genital organ	9 (13.2)
Made to touch and fondle genital or other body parts	29 (42.6)
Made to watch sexual activities	45 (66.2)
Forced to involve in sexual intercourse	3 (4.4)
Other	4 (5.9)
**Frequency of CSA** ^*^	
1 time	29 (45.3)
2-5 times	13 (20.3)
More than 5 times	4 (6.2)
Don't Remember	18 (28.1)
**Place of CSA** ^*^	
Home	23 (39.7)
School	10 (17.2)
Public places	28 (48.3)
Bus	9(14.0)
Others	2(3.1)
**Immediate effect** ^*^	
Scared	16 (26.2)
Danger	16 (26.2)
Ashamed	23 (37.7)
Worried	15 (24.6)
**Disclosure of CSA**	
Yes	35 (54.6)
No	29 (45.3)
**Disclosed with (n = 35)**	
Parents	2 (5.7)
Friends	33 (94.3)
**Response to Disclosure (n = 35)**	
Believed by none	12 (32.4)
No one cared	13 (38.2)
Advised not to share with anyone	7 (20.6)
Needful help was provided	3 (8.8)
**Reason for not Disclosing(n = 35)**	
Didn't realized it as wrong deeds	7 (13)
Because of shamefulness	24 (44.4)
Assumed not to be believed by other	10 (18.5)
Thought it as normal	13 (24.0)

* Indicate multiple response question

Out of the total respondents (n = 405) participated in the survey, 291 (71.9%) children were of age group 1015 years. Both gender, male 223 (55.1%) and female 182 (44.9%) were almost equal in number. About 234 (57.8%) students belonged to a joint family. Maximum participants 128 (31.6%) had family income less than Rs 20,000 (Table 2).

**Table 2:** Demographic Information (n = 405).

Demographic information	n (%)
**Age Category**	
10-15	291 (71.9)
16-20	114 (28.1)
**Gender**	
Male	223 (55.1)
Female	182 (44.9)
**Family Type**	
Nuclear	171 (42.2)
Joint	234 (57.8)
**Family Income**	
Less than 20000	128 (31.6)
20000-30000	124 (30.6)
30000-40000	59 (14.6)
40000-50000	42 (10.4)
More than 50000	52 (12.8)

* Indicate multiple response question

Maximum participants 340 (85.9%) had a good understanding of CSA. Out of total, 41 (10.4%) understood CSA as an act of making children touch and fondle genital or other body parts. 185 (72.5%) of participants received child sexual abuse related information via teachers followed by elder people 93 (36.5%). One third (31%) of children received information from their parents and 33.7% of them gained it from friends. Out of 79 respondents, 58 (73.42%) of them received CSA related information by their mothers (Table 3).

**Table 3:** Knowledge and source of CSA (n= 405)

Understanding on CSA^*^	n (%)
To force to watch or show genital organ	6 (1.5)
To make children touch and fondle genital or other body parts	41 (10.4)
To make children watch sexual activities	3 (0.8)
To force children to involve in sexual intercourse	18 (4.5)
All	340 (85.9)
**Source of information** ^*^	
Parents	79 (31.0)
Teachers	185 (72.5)
Elder People	93 (36.5)
Friends	86 (33.7)
All	9 (3.5)
**Sharing of information by parents** ^*^	
Father	6 (7.5)
Mother	58 (73.4)
Both	15 (18.9)

* indicate multiple response question

Majority 42 (65.63%) of the predators were male and 39 (60.1%) of them were in the age group of less than 20 years. More than half of the predators 33 (54.1%) were stranger to the victim, followed by neighbors 21 (29.6%) (Table 4).

**Table 4:** Characteristics of predator (n = 64).

Predator	n (%)
**Gender**	
Female	22 (34.4)
Male	42 (65.6)
**Age**	
Less than 20	39 (60.1)
More than 21	25 (39.9)
**Relation** ^*^	
Father/Mother	1 (1.6)
Neighbor	21 (29.6)
Brother/Sister	3 (4.9)
Relatives	4 (6.6)
Teacher	2 (3.3)
Stranger	33 (54.1)

* indicate multiple response question

Half of respondents 212 (52.3%) used social media sites and about 11 (5%) of the respondents were sexually abused via them.

## DISCUSSION

WHO and UNICEF have identified child abuse as a global public health concern.^13^ Our study had higher prevalence of CSA among boys (73.43%) as compared to girls (26.56%). Prevalence was also higher in males (54.8%) in a study conducted by Rajbanshi in Nepal among 13-15 years high school students of Kathmandu valley.^10^ The reason for higher prevalence among boys might be their openness to share about sexual issues compared to girls and higher peer pressure to watch sexual media contents.^14^’^15^

Rajbanshi, in his study “Prevalence of Sexual Abuse among School Children” carried out in selected high schools in Kathmandu Valley in 2012, found that out of total 150 student respondent 41.3% of them had faced any sort of sexual abuse either verbal, exhibitionism, or body contact.^10^ But in contrast, our study suggested that, there was prevalence of 15.8% of CSA. The reason for comparatively less prevalence in this study was the difference in definition of CSA. Verbal form of CSA was not included as sexual abuse in this study. Rajbanshi also found that they were mostly abused by their own family members and relatives rather than strangers which coincided with our study that found out 54.1% of predators as stranger. But we could not ignore the fact that children were found to be abused by their neighbor and their own family members as well which was a matter of concern. Similarly, a retrospective and descriptive analysis of cases of sexual abuse victims examined in the forensic medicine department at IOM, Maharajgunj and Gandaki Medical College, Pokhara over four years (2012-2016 A.D.) provided the fact that 87% of the perpetrator were known individuals and only 13% were strangers.^16^

A systematic review done among studies published between 2002-2009 reported that 9 girls and 3 boys out of 100 were victims of forced intercourse.^17^ We had a total of three respondents who were victims of forced sexual intercourse. In cases, such as CSA with lasting effect on the victim, even 3 is a big number.

Our study showed that most respondents were abused between the ages of 12 and 16 which matches with the results from sexual abuse victims examined in the forensic medicine department of different Nepali Medical Schools where half of the victims were from this age group.^16^ Many of our respondents claimed to have forgotten the incident that might have happened before the age of 11. In a study from southern Brazil, 60% of all reported CSA happened before age 12.^18^ All these studies revealed that CSA is mostly observed in the beginning phase of puberty. So, special concern for children is needed at that particular stage of life. At this age children are going through many changes physically and mentally. According to Hall, this is a storm and stress period meaning a time marked by psychological characteristics of contradiction and conflict. They are vulnerable and as the predators are usually known and trusted caregivers' children often fall victim to sexual abuse.^19^

The prevalence of forcing children to watch and show genitals in our study was 13.2%. This number matches with that of a study from South India done among college students (10%).^20^

A large-scale study conducted in Sweden revealed that the disclosure rate among sexually abused girls was 81% and boys was 69% among high school seniors. They most often disclosed with friends of the same age and few with professionals and authorities.^21^ In our study the victims disclosed incidents mostly with friends (94.3%). A survey conducted in Finland showed that the most of the children (80%) had disclosed to someone, most commonly friends (48%), adults (26%), and authorities (12%). The major reason for not disclosing was that they didn't consider the experience serious enough for reporting (41%); half of the children having CSA experiences did not selflabel their experiences as sexual abuse and 14% lacked the courage to disclose.^22^ In this study, victims didn't disclose the problem due to shamefulness (44.4%), considered it was normal (24.07%).

## CONCLUSIONS

Respondents had faced child sexual abuse in different forms and majority were forced to watch sexual activities. However, the disclosure rate was very low. Children rarely reported sexual harassment directly after the incident. In addition, disclosure appears to be a phase rather than a discrete episode and is often triggered after a physical complaint or a behavior change. The reluctance to disclose abuse tends to stem from a fear of the perpetrator. So, there is still a need for an awareness program on CSA to students, parents and other community people as well. The conducive and friendly environment should be created where the children can freely express what they have felt and experienced. This may help them to disclose the suffering they were passing through.
